# The Musashi RNA binding proteins direct the translational activation of key pituitary mRNAs

**DOI:** 10.1038/s41598-024-56002-8

**Published:** 2024-03-11

**Authors:** Jewel Banik, Ana Rita Silva Moreira, Juchan Lim, Sophia Tomlinson, Linda L. Hardy, Alex Lagasse, Anessa Haney, Meghan R. Crimmins, Ulrich Boehm, Angela K. Odle, Melanie C. MacNicol, Gwen V. Childs, Angus M. MacNicol

**Affiliations:** 1 Department of Neurobiology and Developmental Sciences, University of Arkansas for Medical Sciences, 4301 W Markham, Slot 814, Little Rock, AR 72205 USA; 2 Arkansas Children’s Nutrition Center, Arkansas Children’s Hospital, Little Rock, AR USA; 3 Department of Experimental Pharmacology, Center for Molecular Signaling, Saarland University School of Medicine, Homburg, Germany

**Keywords:** Translation, Molecular neuroscience

## Abstract

The pituitary functions as a master endocrine gland that secretes hormones critical for regulation of a wide variety of physiological processes including reproduction, growth, metabolism and stress responses. The distinct hormone-producing cell lineages within the pituitary display remarkable levels of cell plasticity that allow remodeling of the relative proportions of each hormone-producing cell population to meet organismal demands. The molecular mechanisms governing pituitary cell plasticity have not been fully elucidated. Our recent studies have implicated a role for the Musashi family of sequence-specific mRNA binding proteins in the control of pituitary hormone production, pituitary responses to hypothalamic stimulation and modulation of pituitary transcription factor expression in response to leptin signaling. To date, these actions of Musashi in the pituitary appear to be mediated through translational repression of the target mRNAs. Here, we report Musashi1 directs the translational activation, rather than repression, of the *Prop1, Gata2* and *Nr5a1* mRNAs which encode key pituitary lineage specification factors. We observe that Musashi1 further directs the translational activation of the mRNA encoding the glycolipid Neuronatin (*Nnat)* as determined both in mRNA reporter assays as well as in vivo. Our findings suggest a complex bifunctional role for Musashi1 in the control of pituitary cell function.

## Introduction

The pituitary serves as a master endocrine gland and secretes hormones critical to control reproduction, growth, metabolism and responses to stress^1,2^. The anterior pituitary produces key hormones from specialized secretory cell types: Follicle-stimulating hormone (FSH) and Luteinizing hormone (LH) from gonadotropes, Growth hormone (GH) from somatotropes, Adrenocorticotropin (ACTH) from corticotropes, Thyroid-stimulating hormone (TSH) from thyrotropes and Prolactin (PRL) from lactotropes. Pituitary cell plasticity has been defined classically by the capacity of a pituitary hormone-producing cell type to increase cell numbers, hormone stores, and/or size in response to a physiological stimulus or need^3–8^. In the short term, changes in pituitary hormone output are mediated through hypothalamic regulation and peripheral target organ feedback, but over extended periods of altered demand, functional adaptation is required at the level of hormone-producing cell populations within the pituitary. Several non-mutually exclusive processes have been proposed to underlie pituitary plasticity including recruitment and differentiation from the adult stem cell population resident in the pituitary, proliferation of existing hormone-producing cells and/or transdifferentiation of one hormone-producing cell type to another^4,9–16^. Stem cell ablation experiments suggest that these cells are only recruited in cases of catastrophic injury^17^, although recent studies have demonstrated a role for stem cells as a paracrine signaling hub^18^ that may regulate pituitary plasticity of the hormone cell lineages^19^. The molecular mechanism(s) underlying pituitary hormone cell lineage plasticity in response to organismal demand remain poorly characterized but likely involve both transcriptional and post-transcriptional regulation.

The Musashi family of sequence-specific mRNA translational control proteins have been shown to modulate translation of target mRNAs that are required for pituitary hormone production and gonadotrope function^20–22^. The Musashi RNA binding proteins are evolutionarily conserved, sequence-specific regulators of stem cell fate. In vertebrates, two paralogs have been identified, Musashi1 (*Msi1*) and Musashi2 (*Msi2*). The proteins encoded by both genes have been extensively studied in stem cells where they play a necessary role in maintaining stem cell self-renewal and opposing differentiation and appear to function in a redundant manner^23–29^. The Musashi1 protein has been characterized as a mRNA translational repressor of neural cell fate. Binding of Musashi1 to the regulatory 3′ untranslated region (3′ UTR) of the mRNAs encoding the Notch signaling inhibitor endocytic adaptor protein (*Numb)* and cyclin-dependent kinase (CDK) inhibitor, (*p21*^*WAF-1*^) results in repression of the translation of their respective proteins in vitro and in vivo^30,31^. Our group was the first to report that Musashi1 and Musashi2 could conversely direct translational activation of target mRNA translation in a context-dependent manner^32^. The ability of Musashi to translationally activate target mRNAs was shown to be dependent on the phosphorylation of two conserved sites within the C-terminal domain of both Musashi1 and Musashi2^25,33^.

In addition to expression in adult tissue pituitary stem cells, Musashi was unexpectedly found to be highly expressed in mature hormone-producing cell lineages of the anterior pituitary, and pituitary expression levels of *Msi1* and *Msi2,* are second only to the expression seen in gonads^20^*.* We have shown that Musashi exerts translational repression on the POU1F1 lineage specification transcription factor*,* Gonadotropin releasing hormone (GnRH) receptor* (Gnrhr),* Prolactin (*Prl),* Thyroid stimulating hormone-beta *(Tshb)* and Follicle stimulating hormone-beta* (Fshb)* mRNAs as demonstrated through reporter assays, and recently, we have reported that the *Gnrhr* and *Fshb* mRNAs are direct targets and subject to Musashi-dependent repression during estrous cycle gonadotrope remodeling in vivo^20–22,34^.

A recent Musashi RNA-immunoprecipitation sequencing (RIPseq) study identified 1184 pituitary mRNAs that interact specifically with Musashi, suggesting a broad role for Musashi action in modulating pituitary function^22^. Independent qPCR validation confirmed in vivo Musashi1 and Musashi2 interaction with a cohort of these target mRNAs including *Fshb,* the Prop Paired-like homeobox 1 (*Prop1),* GATA binding factor 2 *(Gata2)* and Nuclear receptor subfamily 5 group A member 1 *(Nr5a1)* lineage specification transcription factors and also Neuronatin (*Nnat),* which encodes the highly expressed glycolipid^22^. Here, we have examined the functional consequence of Musashi regulation of these target mRNAs and demonstrate that unlike prior Musashi1 target pituitary mRNAs (*Gnrhr, Pou1f1, Tshb, Prl* and Fshb), Musashi1 directs the translational activation of the *Prop1, Gata2, Nr5a1* and *Nnat* mRNAs in reporter assays. Using a mouse model where both *Msi1* and *Msi2* are specifically deleted in pituitary gonadotropes, we further demonstrate that the *Nnat* mRNA is a target of Musashi regulation in vivo*.* Taken together, our findings indicate that Musashi exerts differential, target mRNA-specific regulation within the mouse pituitary.

## Results

### Musashi1 directs mRNA translational activation via the Prop1 3′ UTR

**Figure 1:**
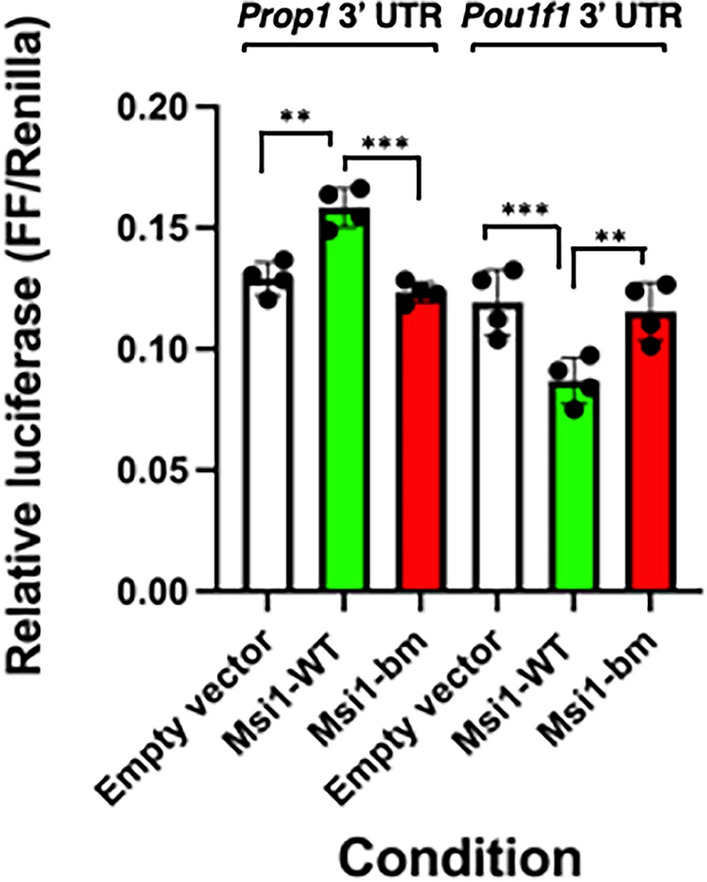
Musashi directs translational activation via the *Prop1* mRNA 3′ UTR. NIH3T3 cells were co-transfected with the full length pmiRGLO *Prop1* 3′ UTR plasmid or the 552 bp *Pou1f1* 3′ UTR Firefly luciferase reporter plasmid and either a plasmid encoding the eGFP moiety alone (peGFPN1), or eGFP tagged forms of wild-type Musashi1 (Msi1-WT) or an RNA binding mutant form of Musashi1 (Msi1-bm). Firefly luciferase values were normalized to the expression of a control Renilla luciferase expressed from the same plasmid (FF/Renilla). Values that differ significantly after one-way ANOVA F(5, 18) = 23.42 are indicated, **(*p* < 0.01) or ***(*p* < 0.001). Representative experiments are shown.

We initially focused our analysis on the potential for regulation of *Prop1* mRNA translation by Musashi, since PROP1 marks a critical progenitor cell population from which all 5 major pituitary hormone-producing cell types are derived^35^ and the *Prop1* mRNA is an in vivo Musashi target^22^. Recently, the full length mRNA for the murine *Prop1* gene has been reported^36^. Analysis of this mRNA sequence revealed a 2247 nucleotide 3′ UTR containing a canonical AATAAA hexanucleotide polyadenylation sequence. We observed that the identified *Prop1* mRNA 3′ UTR contains 24 consensus Musashi binding elements (MBEs, (G/A)U_1–3_AGN^37^), suggesting it may be subject to Musashi-dependent mRNA translational regulation in a manner similar to the other Musashi pituitary targets including the *Gnrhr, Prl, Tshb* and *Pou1f1* mRNAs^20,21,34^ all of which are subject to Musashi-dependent repression, as demonstrated through mRNA reporter assays. To test this directly, the full length murine *Prop1* 3′ UTR was cloned downstream of the firefly luciferase coding sequence and the ability of Musashi1 to exert mRNA translational control was assessed in NIH3T3 cells. NIH3T3 cells lack endogenous Musashi expression and Musashi-dependent mRNA translational repression is dependent upon ectopic expression of *Msi*^37^. When co-expressed with Musashi1 (Msi1-WT), the *Prop1* mRNA 3′ UTR directed significant translational activation of the firefly luciferase mRNA (1.38 fold + /- 0.05 SEM when averaged over 7 independent experiments with mean fold activations of 1.44, 1.22, 1.23, 1.43, 1.55, 1.38 and 1.48) (Fig. 1). By contrast, and consistent with our prior work^20^, expression of Musashi1 resulted in significant translational repression of a firefly luciferase mRNA under the control of the murine *Pou1f1* mRNA 3′ UTR (37.2% ± 4.1 SEM). For both the *Pou1f1* mRNA 3′ UTR and the *Prop1* mRNA 3′ UTR assay, a mutant Musashi1 protein deficient in RNA binding activity (Msi1-bm) failed to exert translational regulation and was indistinguishable from the empty vector control (Fig. 1).

### Translational activation of the Prop1 3′ UTR reporter is dependent upon Musashi regulatory phsophorylation

**Figure 2:**
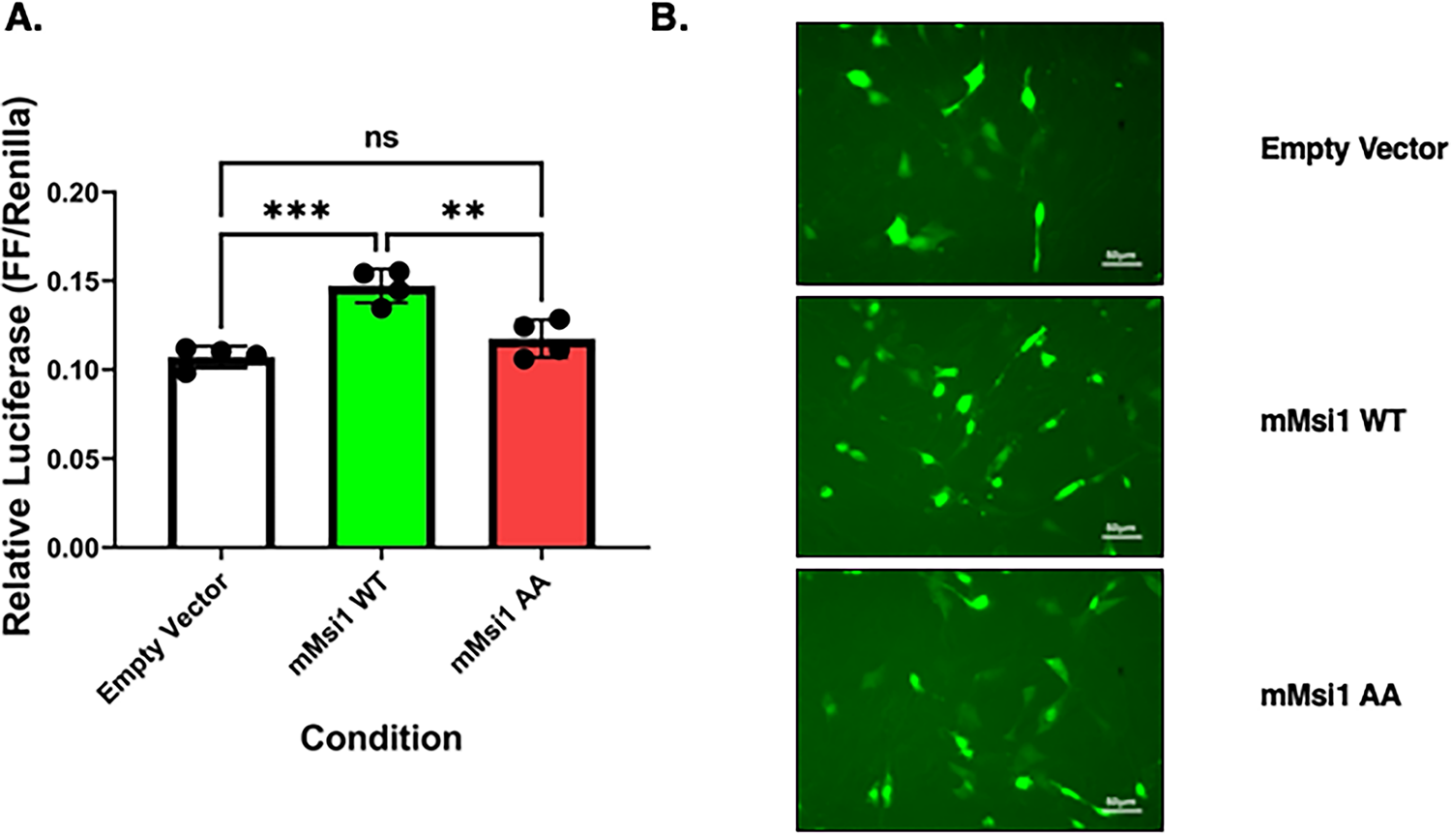
Musashi phosphorylation is required to activate *Prop1* 3′ UTR reporter mRNA translation. (**A**) NIH3T3 cells were co-transfected with the full length pmiRGLO *Prop1* 3′ UTR reporter plasmid and either eGFP tagged forms of mouse wild-type (mMsi1-WT) or mMsi1-AA (encoding a mutant form of MSI1 where the two regulatory sites of activating phosphorylation have been mutated to alanine residues) and the relative Firefly/Renilla luciferase values normalized to the values obtained with same the pmiRGLO plasmid co-transfected with peGFPN1 (Control 3′ UTR). For all panels, values that differ significantly after one-way ANOVA F(2, 9) = 21.46 are indicated, **(*p* < 0.01), or ****(*p* < 0.0001). No significant change between samples is indicated by ns. A representative experiment is shown. **B** Fluorescent microscopy demonstrating relative levels of GFP expression after transfection of empty vector, mMsi1-WT or mMsi1-AA prior to lysing for luciferase assay shown in (**A**).

We have previously observed that Musashi1- and Musashi2-directed translational activation of target mRNAs in *Xenopus* oocyte*s* requires the phosphorylation of two conserved C-terminal serine residues^25,33^. To determine if Musashi1 phosphorylation was similarly required for murine pituitary *Prop1* mRNA translational activation, we repeated our luciferase reporter assay using enhanced green fluorescent protein (eGFP) tagged forms of either wild-type murine Musashi1 (mMsi1 WT) or a mutant Musashi1 where both sites of regulatory phosphorylation were replaced with non-phosphorylatable alanine residues (mMsi1 AA)^38^. As can be seen in Fig. 2A, the ability of wild-type mMsi1 to translationally activate firefly luciferase mRNA translation was lost when the non-phosphorylatable mMsi1 AA mutant protein was employed. eGFP fluorescence in the same transfected NIH3T3 cells used for the luciferase assay (Fig. 2A) confirmed expression of the wild-type mMsi1 and mMsi1 AA proteins (Fig. 2B). We conclude that phosphorylation of Musashi1 is necessary to support translational activation of the reporter mRNA via the *Prop1* 3′ UTR.

### The most distal 195 nucleotides of the Prop1 3′ UTR reporter are sufficient to direct Musashi-dependent translational activation

**Figure 3:**
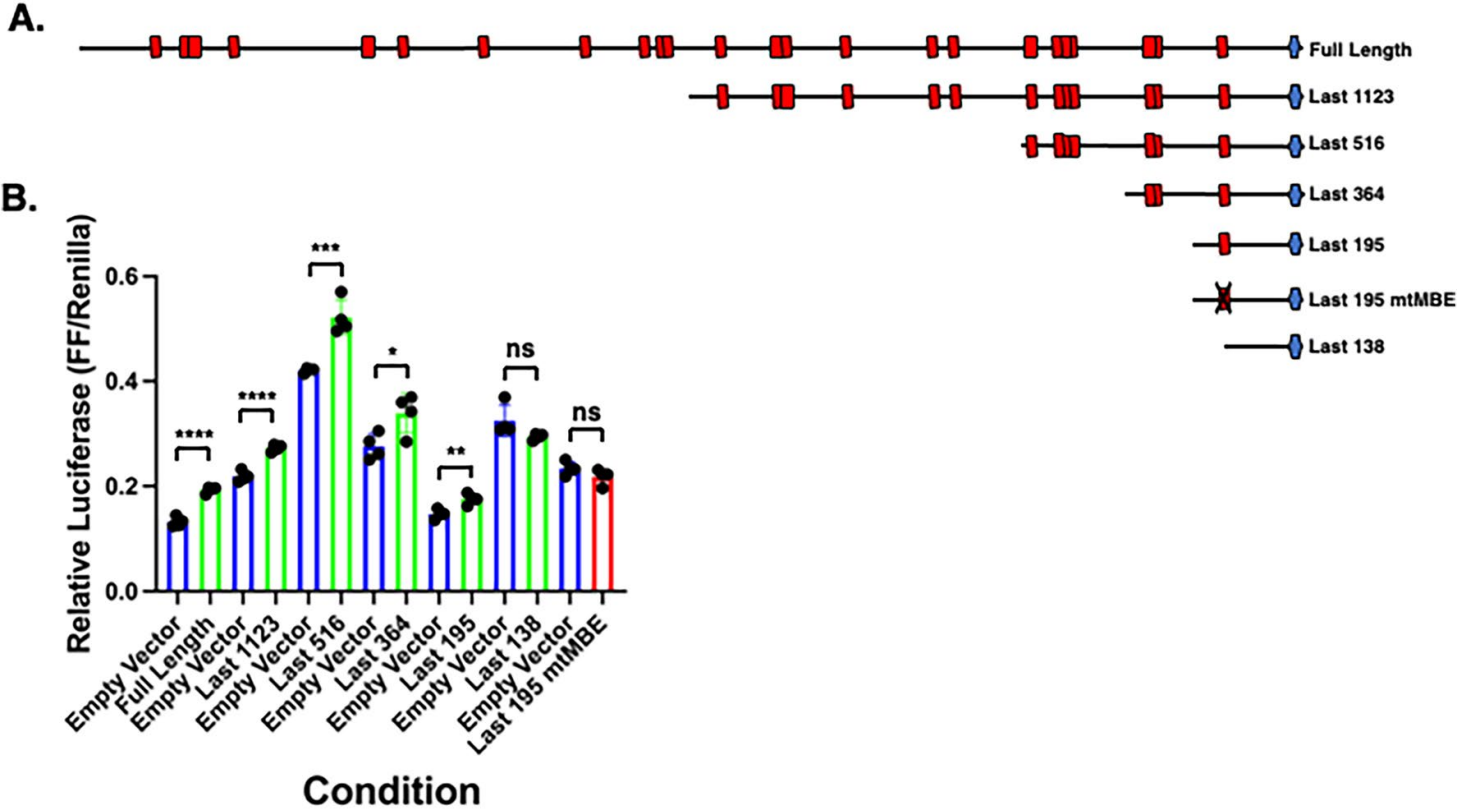
Deletion analysis of the *Prop1* 3′ UTR. (**A**) Schematic representation of the tested pmiRGLO *Prop1* 3′ UTR reporter constructs. Red boxes indicate the position of MBEs, the blue hexagons indicate polyadenylation hexanucleotides and an “X” indicates a mutated MBE. (**B**) NIH3T3 cells were co-transfected with the indicated pmiRGLO full length or deletion *Prop1* UTR reporter plasmid and either empty vector (eGFP) (blue bars) or eGFP tagged Musashi1 (Msi1-eGFP) (green bars) and the relative Firefly/Renilla luciferase values assessed. An MBE mutant form of the last 195 nucleotides was assessed in the presence of empty vector (blue bar) or Msi1-eGFP (red bar). For all indicated pairwise comparisons, values that differ significantly by Student *t* test are indicated, * (*p* < 0.05), ** (*p* < 0.01), *** (*p* < 0.001), or **** (*p* < 0.0001) or ns, not significant. In each case, representative experiments are shown.

To identify MBE(s) that contribute to Musashi-dependent mRNA translational activation (Msi1-eGFP), we generated a series of *Prop1* mRNA 3′ UTR deletion constructs that retain the required 3′ polyadenylation hexanucleotide but are deleted for successively larger proximal portions of the 3′ UTR (Fig. 3A) and assessed their regulatory potential in the firefly luciferase reporter mRNA assay (Fig. 3B). For this experiment, each reporter construct was assessed for translational activation with co-expressed Msi1-eGFP or empty vector (eGFP only) control. All deletion constructs, with the exception of the the last 138 nucleotides of the *Prop1* mRNA 3′ UTR, retained Musashi1-dependent translational activation. Notably the last 138 nucleotides of the *Prop1* mRNA 3′ UTR lacks any MBEs (Fig. 3B, last 138 (NO MBE)).

Since the last 195 nucleotides of the *Prop1* mRNA 3′ UTR retained only one MBE, we reasoned that this MBE was sufficient to direct the observed translational activation. To test this hypothesis, an additional *Prop1* mRNA mutant 3′ UTR construct was generated where the MBE was disrupted within the context of the last 195 nucleotides of the *Prop1* mRNA 3′ UTR (last 195 mutMBE). Mutation of this MBE abolished Musashi1-dependent translational activation directed by the *Prop1* mRNA 195 nucleotide 3′ UTR (Fig. 3B). The necessity of this most distal 3′ MBE within the context of the full length *Prop1* 3′ UTR was not addressed here.

### Musashi exerts mRNA translational activation of Gata2 and Nr5a1 mRNA 3′ UTR reporters

**Figure 4:**
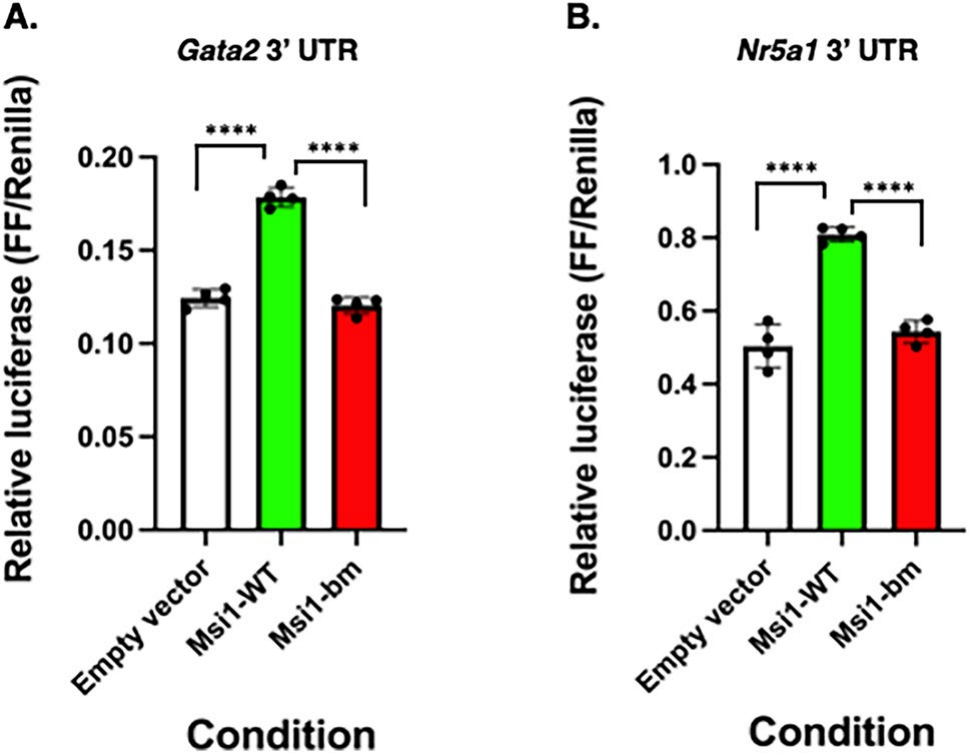
Differential regulation of gonadotrope target mRNAs. NIH3T3 cells were co-transfected with the full length pmiRGLO *Gata2* 3′ UTR (**A**) or *Nr5a1* 3′ UTR (**B**) Firefly luciferase reporter plasmid and either a plasmid encoding the eGFP moiety alone (peGFPN1), or eGFP tagged forms of wild-type Musashii1 (Msi1-WT) or an RNA binding mutant form of Musashi1 (Msi1-bm). Firefly luciferase values were normalized to the expression of a control Renilla luciferase expressed from the same plasmid (FF/Renilla). Values that differ significantly after one-way ANOVA (*Gata2* F(2, 9) = 167.0; *Nr5a1* F(2, 9) = 68.42) are indicated, **** (*p* < 0.0001). Representative experiments are shown.

We are particularly interested in the role of Musashi in mediating the function of the anterior pituitary in control of reproduction^22,34^. Therefore, for our next set of studies, we targeted the mRNAs encoding the transcription factors Steroidogenic factor 1 (SF-1; encoded by the *Nr5a1* mRNA) and GATA2, both of which are crucial for pituitary gonadotrope cell-type specification and function^39,40^ and both of which are pituitary Musashi target mRNAs^22^. The 1536 nucleotide mouse *mGata2* mRNA 3′ UTR contains 5 MBEs and the 1361 nucleotide mouse *mNr5a1* mRNA 3′ UTR contains 8 consensus MBEs. When assessed in mRNA reporter assays, both the *mGata2* mRNA 3′ UTR and the *mNr5a1* mRNA 3′ UTR directed significant Musashi-dependent translational activation (Fig. 4A and B, respectively). This activation was not observed with a mutant Musashi1 disrupted for RNA binding (Msi1-bm) or the empty vector control.

### Identification of the MBEs within the Gata2 3′ UTR that are necessary for Musashi-dependent translational activation

**Figure 5:**
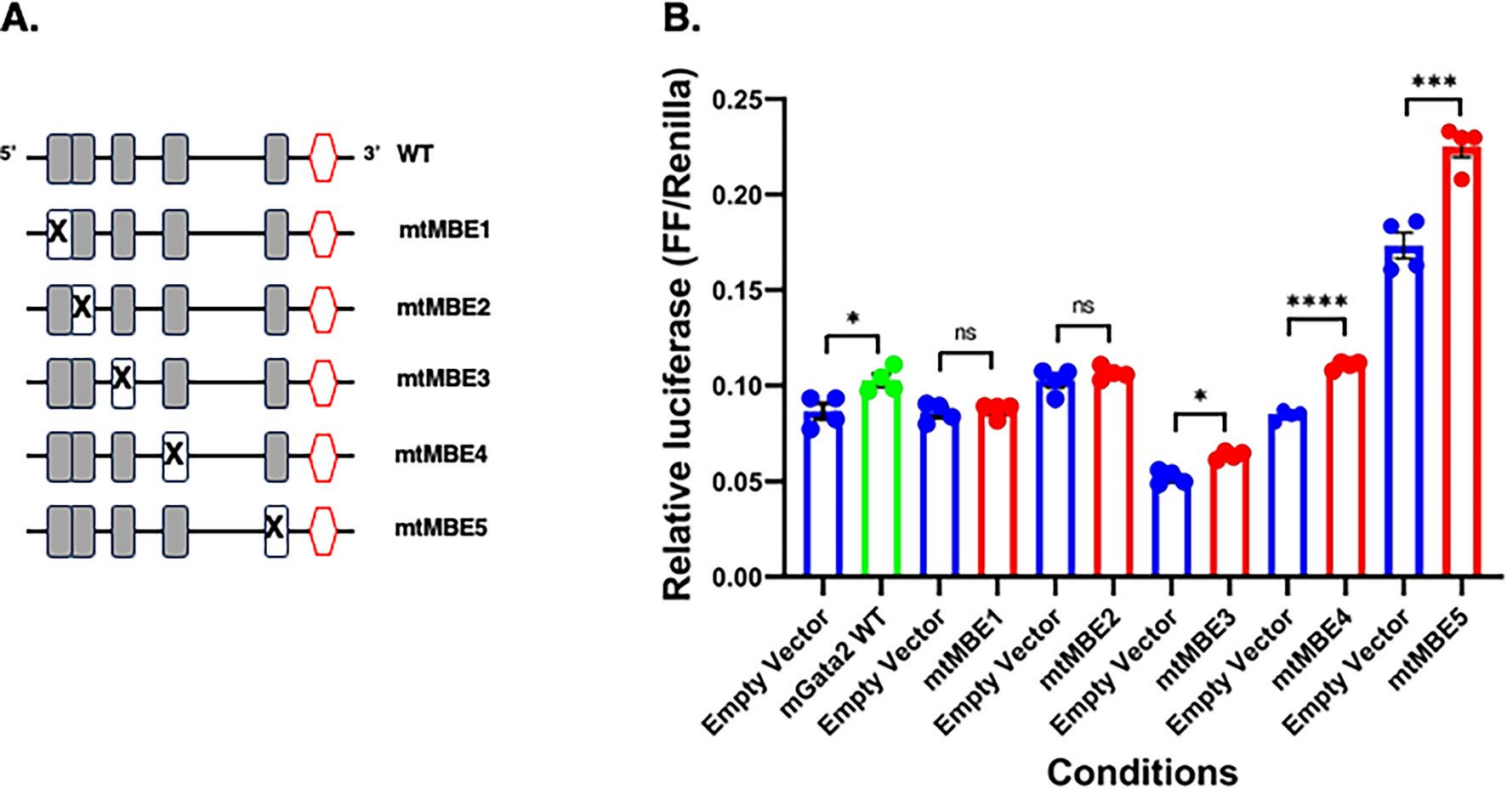
Deletion analysis of the *Gata2* 3′ UTR. (**A**) Schematic representation of the tested pmiRGLO *Gata2* 3′ UTR reporter constructs. Grey boxes indicate the position of MBEs and the red hexagons indicate polyadenylation hexanucleotides. An “X” indicates a mutated MBE within the full length 3′ UTR. (**B**) NIH3T3 cells were co-transfected with the indicated pmiRGLO reporter plasmid and eGFP tagged Musashi 1 and the relative Firefly/Renilla luciferase values compared to the values obtained with same the pmiRGLO plasmid co-transfected with peGFPN1 (Empty Vector). In each case, empty vector controls are shown as blue bars and Msi1-WT co-transfected with wild-type *Gata2* 3′ UTR or mutant *Gata2* 3′ UTRs samples in green or red bars, respectively. For all indicated pairwise comparisons, values that differ significantly by Student *t* test are indicated, *(*p* < 0.05), ***(*p* < 0.001), or ****(*p* < 0.0001) and representative experiments are shown.

Of the 3 transcription factor mRNA targets of Musashi identified in this study (*Prop1, Gata2* and *Nr5a1*), the *Gata2* 3′ UTR has the fewest MBEs. We utilized mutational analysis to identify which *Gata2* 3′ UTR MBE(s) were critical for Musashi-dependent mRNA translational activation. For this experiment, 5 separate reporter constructs were prepared where each contained a disruptive mutation in one of the MBEs (Fig. 5A) and each in turn were compared to the level of translational activation seen with the co-expressed Musashi1 or empty vector control. In this experiment, disruptions of MBE1 or MBE2 abrogated the ability of co-transfected Musashi1 to promote translation of the firefly luciferase reporter mRNA, whereas mutational disruption of MBE3, MBE4 or MBE5 did not prevent reporter mRNA translational activation (Fig. 5B). We conclude that MBE1 and MBE2 are essential for Musashi-dependent activation via the *Gata2* 3′ UTR as disruption of either abrogates Musashi function.

### The Nnat mRNA is a target of Musashi-dependent mRNA translational activation

**Figure 6:**
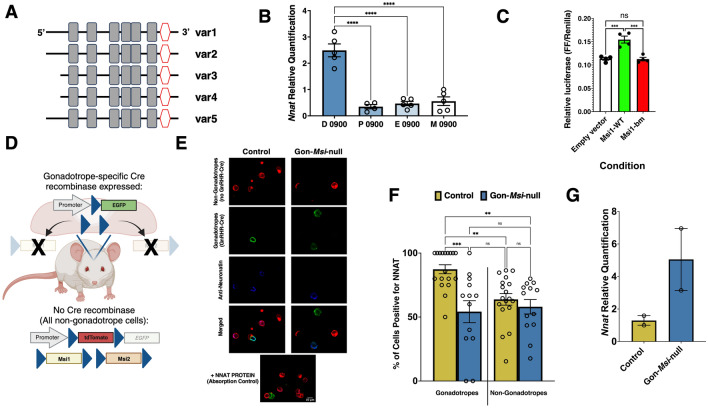
NNAT is a novel in vivo MSI target in gonadotropes. (**A**) A schematic representation of the five *Nnat* mRNA variant 3′ UTRs. Grey boxes indicate the position of MBEs and the red hexagons indicate polyadenylation hexanucleotides. (**B**) *Nnat* mRNA levels in whole pituitaries of adult control females in the morning (0900) of diestrus (D), proestrus (P), estrus (E) and metestrus (M). For each stage, n = 4–5 pituitaries. Relative quantification results are shown, and differences were determined by One-Way ANOVA (F(3, 15) = 2.11) followed by Tukey’s multiple comparisons test. Relative quantification was performed using non-pituitary cell lysate with similar abundance of both *Ppia1* and *Nnat* transcripts*.* (**C**) Firefly luciferase reporter assay in NIH/3T3 cells co-transfected with the pmiRGLO *Nnat* 3′ UTR plasmid and either the eGFP moiety alone (Empty vector), or eGFP tagged forms of the wild-type Musashi1 (Msi1-WT) or an RNA binding mutant of Musashi1 (Msi1-bm). Firefly luciferase values were normalized to the expression of a control Renilla luciferase expressed from the same plasmid (FF/Renilla). The graph is representative of 3 separate experiments, with each condition measured in quadruplicate. Differences were determined by One-Way ANOVA F(2, 9) = 4.77) followed by Tukey’s multiple comparisons test. (**D**) Schematic representation of the reporter and floxed Musashi transgenes in the presence/absence of Cre recombinase expression. In control animals, no Cre-recombinase is expressed, and Musashi is expressed normally. All cells in the body fluoresce red (tdTomato). In Gon-*Msi1/2-*null animals, the same is true *except* for the gonadotropes. The GnRHR-IRES-Cre drives Cre expression specifically in gonadotropes. In these cells only, *Msi 1* and *Msi2* are excised, as is tdTomato. EGFP is expressed only in these gonadotropes. (**E**) Fluorescent immunolabeling of NNAT (blue) in Gon-*Msi*-null gonadotropes (green) vs non-gonadotrope pituitary cells (red). An absorption control, in which NNAT protein was added shows the specificity of the antibody. (**F**) At least 200 cells were analyzed per animal, and three animals are represented within each genotype. The proportion of cells labelled for NNAT was determined, and statistical differences were calculated using One-Way ANOVA (F(3, 58) = 1.45) followed by Tukey’s multiple comparisons test. (**G**) qPCR quantification of *Nnat* mRNA levels (relative to *Ppia*) in duplicate samples from control or Gon-*Msi-*null diestrous female pituitaries as indicated. Each experimental group has n = 2 pituitary cell pools, with n = 3 pituitaries/pool (no statistical analysis performed due to limited n). For all figures, **p < 0.01, ***p < 0.001, ****p < 0.0001. Panel D was created with Biorender.com.

In addition to these characterized lineage specification transcription factors, our Musashi RIPseq analyses revealed a number of endogenous pituitary mRNA targets, including the highly expressed *Nnat* mRNA, encoding the developmental proteolipid Neuronatin^22^. The mouse *Nnat* mRNA has five variants. The mRNA regulatory 3′ UTR is 897 nucleotides long and identical in variants 1, 2 and 5 (RefSeq Accession: NM_010923.3, NM_180960.3, and NM_001291130.1, respectively) and is 760 nucleotides long in variants 3 and 4 (RefSeq Accession: NM_001291128.1, and NM_001291129.1, respectively). Variants 3 and 4 have identical 3′ UTRs and represent a truncated form of *Nnat* 3′ UTR, as they lack the first 137 nucleotides from the 5′ end of variants 1, 2 and 5. Notably, all the variants contain the same 6 consensus MBEs (Fig. 6A). The expression of *Nnat* in gonadotropes has not been previously studied and so we assessed the levels of pituitary *Nnat* transcript during the four different stages of the estrous cycle in control females. We found that *Nnat* mRNA expression is highest in the morning of diestrus compared to all other stages (*p* < 0.0001) and remains at basal levels for the remainder of the estrous cycle (Fig. 6B, Mean RQ ± SEM: Diestrus: 2.49 ± 0.245, Proestrus: 0.348 ± SEM 0.083, Estrus: 0.467 ± 0.081, Metestrus: 0.556, ± 0.165). To evaluate if Musashi regulates translation of the *Nnat* mRNA, a luciferase reporter mRNA assay was conducted. Figure 6C shows that the co-expression of Musashi1 activates *Nnat* 3′ UTR reporter mRNA translation by 1.29 fold (129 ± 3%, *p* < 0.005; average activation from 3 independent experiments). The activation of *Nnat* mRNA translation was not observed with a mutant Musashi1 disrupted for RNA binding (Msi1-bm) or the empty vector control.

We have recently reported a mouse model where *Msi1* and *Msi2* are selectively deleted within the pituitary gonadotrope population (Gon-*Msi*-null). This mouse model also incorporates a CRE reporter^22^ such that gonadotropes are lineage traced through expression of green fluorescent eGFP while the other cell populations in the pituitary express the red fluorescent tdtomato marker (Fig. 6D). We determined if neuronatin (NNAT) protein levels were specifically altered in mouse gonadotropes lacking Musashi1 and Musashi2. The dependence of *Nnat* mRNA translation in vivo upon Musashi was determined by immunolabeling of NNAT protein in control and Gon-*Msi-*null female diestrous pituitaries (Fig. 6E). We confirmed that in control mice, NNAT is expressed in both gonadotrope and non-gonadotrope pituitary cell populations. By contrast, in the absence of gonadotrope Musashi, the total number of gonadotropes (green cells) with detectable labeling for NNAT was reduced by 25% (Fig. 6F, Control: 87.35% ± SEM 3.518, Gon-*Msi-*null: 54.23% ± SEM 8.583, *p* < 0.0005), despite the fact that *Nnat* mRNA levels trended much higher in the pituitary of the diestrous Gon-*Msi-*null female mice (Fig. 6G, dispersed whole pituitaries). These results are consistent with a role for Musashi in promoting NNAT levels. No significant reduction in NNAT levels were seen in the non-gonadotrope pituitary population (Fig. 6F, Control: 63.78% ± SEM 4.517, Gon-*Msi-*null: 58.03% ± SEM 5.622), confirming the specific requirement for Musashi in support of NNAT protein levels in the gonadotrope population^22^.

### Pituitary mRNA 3′ UTR MBE distribution and motif utilization

**Figure 7:**
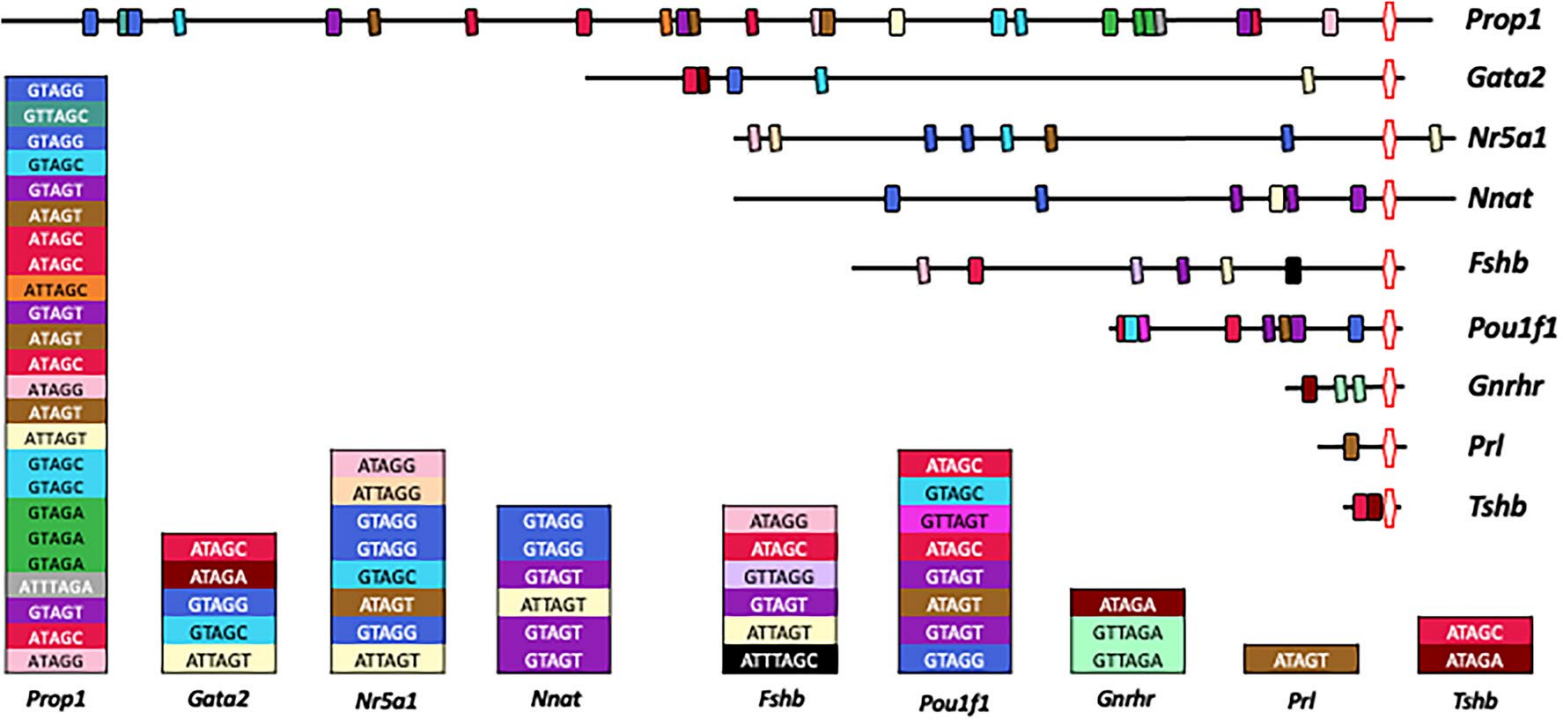
Pituitary mRNA 3′ UTR MBE distribution and motif utilization. Schematic representation of indicated pituitary mRNA 3′ UTRs (upper) and MBE motif distribution (lower). Boxes indicate the position of MBEs and the polyadenylation hexanucleotide is shown as a red bordered hexagon. The upper four 3′ UTRs are activated by Musashi, the lower five are repressed. Length of the black line is proportional to length of the indicated 3′ UTR. Boxes are color coded by MBE motif sequence and are stacked top to bottom to indicate 5′ to 3′ MBE distribution along the indicated 3′ UTR.

The molecular determinants that distinguish the previously reported Musashi-mediated target mRNA repression from the Musashi-mediated target mRNA translational activation identified here, are unknown. Alignment of the regulatory 3′ UTR sequences (Fig. 7) of the activated *mProp1, mNr5a1*, *mGata2* and *mNnat* mRNAs with the sequences of the pituitary mRNAs that we have shown to be repressed for translation (*mFshb, mGnrhr, mPou1f1, mPrl* and *mTshb*), revealed no obvious differences in MBE position within the mRNA 3′ UTRs, MBE density on the mRNA 3′ UTRs, MBE proximity to the STOP codon or polyadenylation hexanucleotide or sequence preference within the MBE consensus motifs^22^.

## Discussion

In this study we report the identification of four pituitary mRNAs that are bound by Musashi in vivo and which are subject to Musashi-dependent translational regulation when assessed in mRNA reporter assays. In contrast to the pituitary mRNA targets that we have previously characterized (*Gnrhr, Fshb, Pou1f1, Prl* and *Tshb* mRNAs^20–22,34^) which are repressed by Musashi, the *Prop1, Gata2, Nr5a1* and *Nnat* mRNA 3′ UTRs direct Musashi-dependent mRNA translational activation. Consistent with the findings from the reporter assay, we demonstrate that levels of NNAT protein are decreased when Musashi is deleted specifically within pituitary gonadotropes, consistent with a requirement for Musashi to promote translation of the *Nnat* mRNA in vivo*.*

The pituitary master transcription factor, PROP1 is initially expressed from embryonic days 11.5 to 14.5 and activates pathways that lead to the differentiation and expansion of all five hormone-producing lineages^35,41–45^. PROP1 activates transcription of the gene encoding POU1F1 (also known as PIT1), which is essential for the maturation of somatotrope, lactotrope and thyrotrope lineages through transactivation of the genes encoding GH, GH releasing hormone receptor (GHRHR)*,* PRL, and TSHβ^35,42–44,46–49^. Our findings indicate that the *Prop1* mRNA 3′ UTR and the *Pou1f1* mRNA 3′ UTR are subject to opposite mechanisms of translational control by Musashi, when assessed within the same cellular context (NIH3T3 cell reporter assay). Musashi1 directs the translational activation of reporter mRNAs under the control of the *Prop1* 3′ UTR, whereas Musashi1 exerts translational repression of reporter mRNAs under the control of the *Pou1f1* 3′ UTR (Fig. 1). While Musashi was originally identified as a repressor of target mRNA translation, prior work from our laboratory demonstrated that Musashi could activate target mRNA translation in a cell context-dependent manner^32,50,51^. In the current study the NIH3T3 cells were transfected in parallel from the same source dish and cultured in an identical manner, indicating that the differential control of translational activation *versus* repression is due to the distinct input mRNA 3′ UTRs rather than cellular context per se.

We have recently implicated Musashi as a regulatory player in gonadotrope remodeling and cell plasticity^22,52^. The phenotypic plasticity of the gonadotrope population is critical for the normal progression of menstrual cycles (human) and estrous cycles (rodent) to support ovulation and follicle maturation via the tight regulation of LH and FSH surges. In preparation for the midcycle LH/FSH surge and the secondary rise in serum FSH^53–59^, gonadotropes respond to increased hypothalamic GnRH pulses^60^ which favor FSH release. FSH stimulates ovarian follicles to produce estradiol, which exerts an indirect positive feedback effect on GnRH neurons and a direct positive feedback effect on gonadotropes^61^, including stimulation of second messengers involved in production of GnRHR and gonadotropins^62–67^. More rapid GnRH pulses then evoke the midcycle LH surge^59^ which is important for the luteinization of the follicle and the functional maturation of the oocyte nucleus^68^. The gonadotrope population remodeling for the next cycle begins with increases in levels of *Lhβ* mRNA during the surge^56–59,69–72^ and *Fshβ* mRNA during its estrous rise^69–71,73^. *Lhβ* and *Fshβ* mRNA levels then decline to a nadir during estrus and metestrus^57,58,69,71^. The discovery that Musashi binds and inhibits translation of *Gnrhr* mRNA in vitro compelled us to create an in vivo gonadotrope-specific Musashi deletion model to define the role of Musashi in gonadotrope remodeling.

Female mice with deletion of *Msi1/2* selectively in gonadotropes show altered levels of GnRHR, FSH and LH proteins but appear to cycle normally^22^. Female mice have significantly elevated GnRHR levels in diestrus and elevated pituitary stores of FSH early in estrus along with higher serum FSH levels. These findings correlate well with the repressive effect of Musashi on GnRHR and FSH protein levels^22,34^. The rapid rise in LHβ content in control mice from diestrus to proestrus afternoon is blunted in mice with *Msi*-null gonadotropes. However, the *Lhb* mRNA contains no consensus MBEs in the mRNA 3′ UTR and *Lhb* is not enriched in pituitary Musashi RIPseq experiments^22^. In this study we report that Musashi exerts translational activation of *Gata2* and *Nr5a1* mRNAs in reporter assays (Fig. 4). Musashi may thus be involved indirectly in promoting *Lhb* mRNA expression by increasing SF-1 and GATA2 levels, which bind to the *Lhb gene* promoter to promote GnRH responsiveness^74,75^. GATA2 is also an important transcription factor for *Cga, Lhb,* and SF-1/*Nr5a1* gene expression^76^. Our findings support a model in which Musashi acts during the estrous cycle to activate translation of the *Gata2* and *Nr5a1* mRNAs to provide SF-1 and GATA2 protein to transcriptionally activate the *Lhb* mRNA to support a midcycle LH surge. Future studies will be necessary to compare SF-1 and GATA2 levels in wild-type and *Msi*-null gonadotropes to validate Musashi-dependent *Gata2* and *Nr5a1* mRNA translational activation in vivo.

The present study also determined that *Nnat* mRNA expression in the pituitary is cycle-dependent and is significantly higher in diestrus than in any other stage of the estrous cycle (Fig. 6). NNAT is a proteolipid involved in neurogenesis in the neonatal brain^77^. In adult humans, the highest *Nnat* gene expression occurs in the pituitary^78^. In the adult mouse pituitary, *Nnat* is the fifth most highly expressed gene^79^. In contrast with the repression of the *Gnrhr* and *Fshb* mRNAs, Musashi binding to the regulatory region of the *Nnat* mRNA promotes translational activation in reporter assays (Fig. 6). Consistent with loss of Musashi-mediated translational activation of the *Nnat* mRNA, cells expressing NNAT protein were decreased by 25% in Gon-*Msi*-null diestrous female mice gonadotropes compared to gonadotropes of control mice (Fig. 6). Future studies will be needed to determine the role that NNAT plays in optimizing gonadotrope function through the cycle.

We have previously reported that the *Gnrhr* mRNA is an endogenous target of Musashi-dependent repression in diestrous female mice^22^ and here we provide evidence that *Nnat* mRNA translation is activated by Musashi in diestrous female mice. Thus, like the identification of distinct mechanisms of Musashi-mediated reporter translation controlled by the *Prop1* and *Pou1f1* mRNA 3′ UTRs in NIH3T3 reporter assays (Fig. 1), Musashi appears to exert opposite mechanisms of regulation of the *Nnat* mRNA (activation) and *Gnrhr* mRNA (repression) mRNAs within the same cellular context (diestrous female gonadotropes) in vivo. Growing evidence suggests similar Musashi target-specific selective repression or activation of translational output within the same cell in both pathological and physiological contexts^80–83^. However, the molecular determinants of differential Musashi target mRNA regulation are unknown.

**Figure 8:**
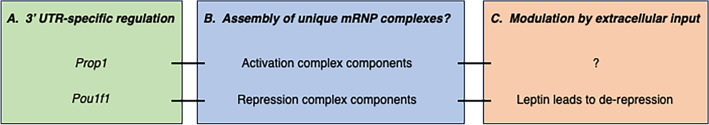
3′ UTR-specific and context dependent mRNA translational regulation by Musashi. Schematic representation of the possible layers of translational control exerted by Musashi. In this model, Musashi interacts with a subset of cellular mRNAs containing MBEs in favorable secondary structure within the 3′ UTR (**A**). Differences in secondary structure or modulation by additional regulatory motifs within each 3′ UTR confer either Musashi-dependent mRNA translational activation (e.g. *Prop1)* or repression (e.g. *Pou1f1*). We hypothesize that each 3′ UTR recruits unique activation or repression proteins to the Musashi mRNP assembled on the target mRNA (**B**). Modulation of the cellular context via extracellular signaling can modulate the behavior of the assembled Musashi mRNPs to alter mRNA translational output (**C**). See text for details.

We have shown that Musashi directs the translational activation of target mRNAs in the *Xenopus* oocyte model system, with both Musashi1 and Musashi2 acting in a functionally redundant manner^32^. The ability of Musashi1 or Musashi2 to direct early class mRNA translation requires progesterone-stimulated regulatory phosphorylation on two conserved serine residues present in both the Musashi1 and Musashi2 proteins^25,33^. Here, we extend these observations from the *Xenopus* system and report that the Musashi-dependent activation of the murine *Prop1* 3′ UTR reporter is ablated when a mutant murine Musashi1 is used that lacks the two sites of regulatory phosphorylation (Fig. 2). These findings are consistent with a requirement for regulatory phosphorylation of Musashi1 to direct translation via the murine *Prop1* 3′ UTR in a manner similar to that seen in progesterone-stimulated *Xenopus* oocytes. The pathways that mediate Musashi phosphorylation in murine pituitaries remain to be determined. Interestingly, in the heterologous NIH3T3 cell mRNA reporter assays, Musashi-dependent repression of the murine *Pou1f1* 3′ UTR and the Musashi-dependent activation of the murine *Prop1* 3′ UTR occurred within the same cellular context. Thus, unlike the *Xenopus* oocyte maturation model where a cell context change in response to progesterone stimulation is required to promote regulatory phosphorylation and Musashi-dependent mRNA activation, in the NIH3T3 cell context, both Musashi-dependent mRNA repression and Musashi-dependent activation occur in parallel. We infer that differences intrinsic to the *Prop1* mRNA 3′ UTR *versus* the *Pou1f1* mRNA 3′ UTR confer Musashi-dependent activation versus repression, respectively (Fig. 8A).

Importantly, while our current findings indicate mRNA 3′ UTR-specific regulation within the same cellular context, we have also observed that extracellular signals can exert control of Musashi function (Fig. 8C). Similar to the ability of progesterone stimulation to modulate Musashi function in *Xenopus* oocytes^32^, leptin stimulation opposes Musashi-dependent repression exerted via the *Pou1f1* 3′ UTR in reporter assays^20^. The effects of leptin in modulation of Musashi-dependent translational activation remain to be determined. Thus, although our data demonstrate that mRNA 3′ UTR-specific determinants are sufficient to dictate repression or activation of Musashi target mRNA translation within a given cellular state, extracellular signaling can further impose additional layers of regulation on Musashi functional control and target mRNA translational output.

Examination of activating *versus* repressing pituitary mRNA 3′ UTRs revealed no clear MBE number, positional dependence, clustering or sequence motif bias^22^ (and Fig. 7). Potential mechanisms that could influence Musashi regulatory function include contributions from 3′ UTR secondary structure^84^ that may influence bound Musashi conformation to favor translational activation or repression of the upstream mRNA open reading frame. In support of the importance of preferred secondary structure for Musashi interactions with target mRNAs ^37,85–87^, over 75% of murine mRNAs contain one or more consensus MBEs in their 3′ UTRs^88^ but only a small proportion of the total pituitary mRNA population (7.3%) exhibited specific and high confidence association with Musashi in an RNA immunoprecipitation analysis^22^. However, while subtle differences in secondary structure could dictate Musashi-dependent repression *versus* Musashi-dependent activation, a recent Musashi2 individual nucleotide resolution cross-linking and immunoprecipitation study found no obvious differences in secondary structure nor accessibility around 3′ UTR cross-link sites for translationally upregulated or downregulated transcripts^80^. An alternative and non-mutually exclusive possibility is that additional regulatory sequence(s) such as miRNA target sites or other RNA binding protein motifs within the mRNA 3′ UTR modulate Musashi binding or activity in a cooperative or antagonistic manner^88^. In either case, mRNA secondary structure and/or additional regulatory motifs likely influence assembly of specific Musashi ribonucleoprotein (mRNP) complexes on the mRNA 3′ UTRs that direct repression *versus* activation of mRNA translation (Fig. 8B). Consistent with the idea of distinct Musashi mRNP complexes, we have reported that Musashi mRNP complexes undergo dynamic remodeling in response to progesterone stimulation of *Xenopus* oocytes^89^. While some Musashi co-associated factors remained constant, other proteins were unique to the Musashi ribonucleoprotein mRNP complex in immature or in progesterone-stimulated, maturing oocytes. Future experiments utilizing proteomic analyses of the Musashi mRNP complexes associating with activated *versus* repressed mRNA 3′ UTRs will help elucidate the molecular determinant(s) dictating Musashi translational outputs as well as their dependence upon extracellular cues.

Together with our earlier work characterizing Musashi-dependent repression of select pituitary mRNAs and an unbiased identification of endogenous mRNA targets of Musashi, the findings of this study show that Musashi is a bifunctional regulator of a broad range of mRNAs within the adult pituitary, exerting control of translational output through either the repression or the activation of distinct mRNA targets. A number of validated Musashi target mRNAs encode transcription factors critical to early pituitary development and hormone-cell type lineage commitment. Furthermore, a number of the proteins encoded by these mRNAs impinge on gonadotrope remodeling during the adult pituitary estrous cycle supporting a requirement for Musashi to mediate cyclic gonadotrope plasticity^22^. Thus, the characterization of Musashi regulation is a promising area for future studies that are relevant to understanding pituitary dysfunction and identification of novel therapeutic targets that control physiological and pathological cell fate transitions.

## Methods

### Animals

All methods are reported in accordance with ARRIVE guidelines (https://arriveguidelines.org). The use of animals was approved by and carried out in compliance with the University of Arkansas for Medical Sciences Institutional Animal Care and Use Committee (IACUC) guidelines. The mice used in these studies were maintained on a 14 h light/10 h dark cycle at 27 °C. The lights are on from 0600 to 2000. All non-breeding mice were fed a standard diet (crude protein ≥ 18%, crude fat ≥ 5%, crude fiber ≤ 5%; LabDiet, 5V5R). All breeder mice were fed a breeder diet (crude protein ≥ 18%, crude fat ≥ 8%, crude fiber ≤ 5%; LabDiet, 5V5M). Food and water were provided ad libitum. Mice were weaned at 21 days of age and housed no more than five animals per cage.

As recently reported^22^, we created a gonadotrope-specific *Msi1* and *Msi2* knockout animal model (Gon-*Msi-*null) by crossing *Msi1* floxed and *Msi2* floxed mice (*Msi1/2*^*flox/flox*^, a gift from Dr. Christopher Lengner) with mice bearing a *Gnrhr*-internal ribosomal entry site (IRES)-Cre (GRIC) driver^90^. Given the potential for extra-pituitary *Cre* expression in males, studies involving the Gon-*Msi-*null line were limited to females^91^. In addition, a floxed fluorescent reporter transgene (mT/mG or *Gt(ROSA)26Sor*^*tm4(ACTB-tdTomato,-EGFP)Luo*^/J, Stock 007,576, *The Jackson Laboratory*) was introduced into the gonadotrope-*Msi*-null mouse line. The addition of the mT/mG construct drives constitutive expression of membrane-targeted tdTomato before Cre excision, and expression of membrane-targeted EGFP after Cre excision^92^. Therefore, all non-gonadotropes express red fluorescence (tdTomato) and all gonadotropes express enhanced green fluorescence (eGFP)^22^. A subset of control animals carrying only GnRHR-IRES-Cre and the floxed reporter transgene were used in immunocytochemistry experiments. All other control females were Cre-negative, with two floxed alleles of the reporter transgene and two floxed alleles of *Msi1/2.* All Gon-*Msi-*null females carried one copy of Cre, two floxed alleles of the reporter transgene and two floxed alleles of *Msi1/2.*

### 3′ UTR cloning

The murine 2247 nucleotide *Prop1* mRNA 3′ UTR (Dr. S.A. Camper) was PCR amplified using primers that added a 5′ NheI site and a XhoI 3′ site, cloned into NheI/XhoI digested pmiRGLO (Clontech) and designated pmiRGLO *Prop1* 3′ UTR. Deletion mutants of the *mProp1* 3′ UTR were generated by Quikchange II PCR mutagenesis (Aligent), inserting a Nhe1 site at the indicated position along the full length 3′ UTR and then re-ligation after digestion with Nhe1 to remove the indicated 5′ region and generate truncated *mProp1* 3′ UTRs of 1123, 530, 364, 195 or 138 nucleotides that retained the 3′ polyadenylation hexanucleotide sequence. The resulting plasmids were designated pmiRGLO 1123 bp, pmiRGLO 530 bp, pmiRGLO 364 bp, pmiRGLO 195 bp or pmiRGLO 138 bp *Prop1* 3′ UTR, respectively. Disruption of the only MBE within the last 195 nucleotide 3′ UTR was performed by Quikchange II PCR mutagenesis (changing the ATAGG motif to AggGG) and designated pmiRGLO 195 bp mutMBE *Prop1* 3′ UTR.

The 3′ UTRs of the murine *Nr5a1* mRNA (NM_001316687) and *Gata2* mRNA (NM_008090) were cloned into the pmiRGLO vector using an identical strategy. In each case, geneblock primers (IDT) were generated for the full length 3′ UTR sequences with the addition of a 5′ NheI and a 3′ SalI restriction site. After digestion of each geneblock primer with NheI and SalI, the recovered fragments were cloned into NheI/SalI digested pmiRGLO. The resulting plasmids were designated pmiRGLO *Nr5a1* 3′ UTR and pmiRGLO *Gata2* 3′ UTR. Disruption of individual MBEs within the full length *Gata2* 3′ UTR was performed by Quikchange II PCR mutagenesis (changing the core TAG sequence within each MBE to ggG) and designated pmiRGLO *Gata2* mtMBE1 (changing the ATAGC motif to AggGC), pmiRGLO *Gata2* mtMBE2 (changing the ATAGA motif to AggGA), pmiRGLO *Gata2* mtMBE3 (changing the GTAGG motif to GggGG), pmiRGLO *Gata2* mtMBE4 (changing the GTAGC motif to GggGC)or pmiRGLO *Gata2* mtMBE5 (changing the ATTAGT motif to ATggGT), respectively.

A construct was also created in which the murine *Nnat* 3′ UTR was cloned into the pmiRGLO plasmid. The 897 bp 3′UTR from the murine *Nnat* variant 1 mRNA (NM_010923.3) was synthesized as a geneblock fragment (IDT) with a 5′ Sac1 site and 3′ Xba1 site and cloned into the Sac1-Xba1 digested pmiRGLO plasmid. The resultant clone placed the *Nnat* 3′-UTR downstream of the FLuc open reading frame and was designated pmiRGLO *Nnat* 3′UTR.

The integrity of all cloned 3′ UTRs was validated by DNA sequencing of the final pmiRGLO plasmids. The pmiRGLO 552 bp *Pou1f1* 3′ UTR plasmid has been previously described^20^.

### Luciferase reporter assays

NIH3T3 cells (ATCC CRL-1658)) were co-transfected with the indicated pmiRGLO 3′ UTR reporter plasmid along with either wild-type murine *MSI1*-eGFP, *MSI1*-bm-eGFP (which has three phenylalanine to leucine mutations within the first RNA recognition motif (RRM1, F63L/F65L/F68L) that attenuates target RNA association^37^), *MSI1*-AA-eGFP (which is mutated to substitute the two sites of regulatory serine phosphorylation to non-phosphorylatable alanine residues), or eGFP (peGFP N1 empty vector control) plasmids as described previously ^20,33,34,38,50^. Expression of the MSI1-eGFP, MSI1-bm-eGFP, and eGFP proteins was confirmed by fluorescence microscopy. Luciferase activity was determined in quadruplicate after 24 h, using the Dual-Luciferase Reporter Assay System (Promega, E2920) and Turner Biosystems luminometer (Promega) according to the supplier's protocol**.** Data are expressed as relative luciferase activity (FLuc/RLuc) in arbitrary units. All experiments were repeated on at least 3 separate occasions.

### Estrous cycle studies

All female mice used for these studies were between 2 and 4 months of age. Vaginal smears were collected daily to identify the stage of the estrous cycle of adult female mice as previously described^93^. Smears were collected daily through two full estrous cycles to ensure all experimental females (Control and Gon-*Msi-*null) were cycling.

Control and Gon-*Msi*-null females were euthanized at 0900 on the mornings of diestrus, proestrus, estrus, and metestrus. Following isoflurane anesthesia and decapitation, whole pituitaries were collected in 150 µL ice-cold Radioimmunoprecipitation (RIPA) buffer (Sigma, R0278) with 10 µg/ml protease inhibitors (ThermoFisher Scientific, 78,425) and homogenized with pellet pestles. From this homogenate, 30 µL was pulled and stored at -20 °C for RNA extraction.

### Quantitative Real-Time PCR

RNA from control and Gon*-Msi*-null pituitary lysates was isolated using the RNAzol extraction method according to the manufacturer’s protocol (Sigma, R4533). The recovered RNA was quantified (Nanodrop One, Thermo Fisher Scientific), and between 100 and 500 ng of RNA was used for synthesis of cDNA using iScript cDNA synthesis kit (Bio-Rad, 1,708,891). The cDNA samples and primers for the transcripts of interest were added to Power SYBR Green PCR Master Mix (Applied Biosystems, 4,367,659) for amplification and detection through qRT-PCR. The qRT-PCR reactions were performed using the QuantStudio 12 K Flex system (Applied Biosystems) with the following three stage protocol: Incubation/Denaturation stage: 50 °C for 2 min, and 95 °C for 10 min; PCR amplification stage (40 cycles): 95 °C for 15 s, 55 °C for 15 s, and 72 °C for 1 min; and Melt Curve stage: 95 °C for 15 s, 60 °C for 1 min, and 95 °C for 15 s. Data collection occurred after each of the 72 °C steps in the amplification stage. Transcripts of interest were normalized to the cyclophilin gene (*Ppia)* expression, and relative expression values were determined by the QuantStudio 12 K Flex Software version 1.0 using the delta delta cycle threshold (cT) method. The housekeeping gene was *Ppia*, and the primers used were: forward 5′-TGGTCTTTGGGAAGGTGAAAG-3′; reverse 5′- TGTCCACAGTCGGAAATGGT-3′. For *Nnat* the primers used were: forward 5′- CTCATCATCGGCTGGTACATC-3′; reverse 5′- ACACCTCACTTCTCGCAATG-3′.

### Pituitary cell dispersion and fixation

Pituitaries from diestrous 0900 female mice were also collected for dispersion, fixation, and immunocytochemistry (ICC). The pituitaries were collected as described above and dispersed as described previously^22^. Following the final wash, the dispersed pituitary cells were resuspended in DMEM + 1:100 insulin–transferrin–sodium selenite (ITS, Sigma, I1884) media supplement + 1:200 protease inhibitor cocktail (Sigma, P8340). The cell count for each sample was determined using a hemocytometer under an inverted light microscope and viability assessed using Trypan blue. Cells were plated on poly-d-lysine coated glass coverslips in 24-well trays, at a density of 16,000 cells per coverslip. The cells were incubated for 45 min to 1 h, to allow adherence to the coverslips. Following incubation, 400 µl of DMEM + ITS + protease inhibitor cocktail was added, and the cells were again incubated for 2 h. The media was removed, and cells were fixed in 4% paraformaldehyde for 30 min at room temperature. The solution was removed and three 15-min washes with phosphate sucrose buffer removed any excess of paraformaldehyde. The phosphate sucrose solution was added to each well containing cells, and the trays were covered with parafilm and stored at 4 °C until used for immunocytochemistry.

### Fluorescent immunocytochemistry of primary pituitary cells

Pituitary cells from fluorescent control and Gon-*Msi*-null female mice in diestrus 0900 were dispersed and fixed as described above. The cells were then immunolabeled for NNAT using a rabbit polyclonal anti-NNAT antibody (Abcam, ab27266). The protocol involved 3 washes in 0.05 M Tris buffer, pH 7.6 followed by 5 min in 0.3% Triton X (Sigma, T8787) and an additional round of 3 washes with 0.05 M Tris buffer. The cells were then treated with blocking solution, containing 10% normal goat serum and 0.1% BSA for 30 min at room temperature, and then were exposed to the anti-NNAT antibody diluted in blocking solution at 1:100, for 30 min at 37 °C in a hybridization incubator with gentle rotation. After this exposure, the cells were washed 3 × with 0.05 M Tris buffer, and incubated in a goat anti-rabbit IgG conjugated to Cy5 (Abcam, ab97077) diluted in blocking buffer at 1:100, for 30 min at room temperature. The cells were once again washed 3 × with 0.05 M Tris buffer and then once with pure H_2_O. The coverslips containing labeled cells were mounted on slides in Vectashield vibrance antifade mounting media without DAPI (Vector Laboratories, H-1700) and imaged using an inverted laser scanning confocal microscope (Zeiss LSM 880 with Airyscan). Cells from 3 animals/genotype were imaged, and at least 200 cells/animal were analyzed. The specificity of this protocol was validated using an absorption control in which 5 µg NNAT peptide (Abnova, H00004826-P01) was incubated overnight at 4 °C with the anti-NNAT antibody. This solution was then applied in the above ICC protocol, and no NNAT signal was observed. For the ICC experiments, the control females were GnRHR-IRES-Cre positive and also carried the reporter transgene (but no floxed *Msi*). This allowed us to have both Control and Gon-*Msi-*null samples wherein all non-gonadotropes fluoresce red and gonadotropes fluoresce green, thus eliminating the need for double immunocytochemistry. The NNAT label is pseudocolored blue to distinguish it from the Cre-reporter fluorescence.

### Statistics

Sample sizes for the animal studies were calculated using post hoc power analyses (alpha = 0.05), and the number of samples/cells evaluated in these studies can be found in the legend for Fig. 6. In vitro tests were repeated at least 3 times. Cell counts, qPCR results, and mRNA reporter assay values were analyzed with Prism statistical software with ANOVA followed by Sidak’s post hoc test unless otherwise noted^94–96^.

## Data Availability

The datasets generated during and/or analyzed during the current study are available from the corresponding author on reasonable request.
